# Multiomics-integrated deep language model enables *in silico* genome-wide detection of transcription factor binding site in unexplored biosamples

**DOI:** 10.1093/bioinformatics/btae013

**Published:** 2024-01-12

**Authors:** Zikun Yang, Xin Li, Lele Sheng, Ming Zhu, Xun Lan, Fei Gu

**Affiliations:** Damo Academy, Alibaba Group, Hangzhou 310023, China; Hupan Lab, Hangzhou 310023, China; Damo Academy, Alibaba Group, Hangzhou 310023, China; Hupan Lab, Hangzhou 310023, China; Damo Academy, Alibaba Group, Hangzhou 310023, China; Hupan Lab, Hangzhou 310023, China; Department of Basic Medical Science, School of Medicine, Tsinghua University, Beijing 100084, China; Tsinghua-Peking Joint Center for Life Sciences, Tsinghua University, Beijing 100084, China; MOE Key Laboratory of Bioinformatics, Tsinghua University, Beijing 100084, China; Department of Basic Medical Science, School of Medicine, Tsinghua University, Beijing 100084, China; Tsinghua-Peking Joint Center for Life Sciences, Tsinghua University, Beijing 100084, China; MOE Key Laboratory of Bioinformatics, Tsinghua University, Beijing 100084, China; Damo Academy, Alibaba Group, Hangzhou 310023, China; Hupan Lab, Hangzhou 310023, China

## Abstract

**Motivation:**

Transcription factor binding sites (TFBS) are regulatory elements that have significant impact on transcription regulation and cell fate determination. Canonical motifs, biological experiments, and computational methods have made it possible to discover TFBS. However, most existing *in silico* TFBS prediction models are solely DNA-based, and are trained and utilized within the same biosample, which fail to infer TFBS in experimentally unexplored biosamples.

**Results:**

Here, we propose TFBS prediction by modified TransFormer (TFTF), a multimodal deep language architecture which integrates multiomics information in epigenetic studies. In comparison to existing computational techniques, TFTF has state-of-the-art accuracy, and is also the first approach to accurately perform genome-wide detection for cell-type and species-specific TFBS in experimentally unexplored biosamples. Compared to peak calling methods, TFTF consistently discovers true TFBS in threshold tuning-free way, with higher recalled rates. The underlying mechanism of TFTF reveals greater attention to the targeted TF’s motif region in TFBS, and general attention to the entire peak region in non-TFBS. TFTF can benefit from the integration of broader and more diverse data for improvement and can be applied to multiple epigenetic scenarios.

**Availability and implementation:**

We provide a web server (https://tftf.ibreed.cn/) for users to utilize TFTF model. Users can train TFTF model and discover TFBS with their own data.

## 1 Introduction

Transcriptional regulation (TR) (Greive and von Hippel 2005) is of great importance for gene expression (Ortega *et al.* 2018) and cell fate determination (Spitz and Furlong 2012, He *et al.* 2014). Many TR functions, such as DNA methylation (Bird 2002), histone modification (Jenuwein and Allis 2001, Li *et al.* 2007), chromatin structure conformation (Almeida *et al.* 2018), have been well studied previously. Transcription Factors (TFs) are proteins that bind to specific regions of DNA to initiate and control gene transcription (Cramer 2019). Numerous high-throughput experiments, such as ChIP-seq (Park 2009), have been conducted to detect transcription factor binding sites (TFBS) (Geertz and Maerkl 2010). with the ENCODE project (The ENCODE Project Consortium 2012) utilizing this technique to investigate around 200 human TFs across nearly 100 human cell lines. Despite these extensive efforts, only a small fraction of all TFs have been identified, prompting researchers to search for improved solutions.

In past decades, remarkable advances of machine learning (ML) and deep learning (DL) methods in computer vision (CV) and natural language processing (NLP) (Hochreiter and Schmidhuber 1997, LeCun *et al.* 2015, He *et al.* 2016, Vaswani *et al.* 2017) have been successfully applied in many academic or industrial fields, including biological sciences (Esteva *et al.* 2019, Baek *et al.* 2021, Elmarakeby *et al.* 2021, Jumper *et al.* 2021, Yang *et al.* 2021). With the hypothesis that TF binds to its sequence-specific nucleotides shorter than 20 base-pairs (bp) (Isbel *et al.* 2022), or motifs (Yáñez-Cuna *et al.* 2012), various computational methods have been developed to predict TFBS (Alipanahi *et al.* 2015, Zhou and Troyanskaya 2015, Kelley *et al.* 2016, Khamis *et al.* 2018, Koo and Ploenzke 2020, Quang and Xie 2020, Zhang *et al.* 2020). Of these, DNABERT (Ji *et al.* 2021), a pretrained model inspired by the popular Bidirectional Encoder Representations (BERT) (Devlin *et al.* 2019) architecture, holds the current state-of-the-art (SOTA) result.

Previous methods often rely solely on DNA sequence to identify TFBS, despite the fact that other variables such as indirect binding of TF to its canonical motif due to protein complex (Gordân *et al.* 2009), epigenetic regulation such as DNA methylation (Zhu *et al.* 2016) and chromatin accessibility (Klemm *et al.* 2019) complicate the binding mechanism (Inukai *et al.* 2017). Moreover, TFBS show cell type-specific (Arvey *et al.* 2012) and stage-specific manner (He *et al.* 2014, Akerberg *et al.* 2019). These additional variables result in TFBS being inconsistent, even across identical DNA sequences (Gulko and Siepel 2019).

Most computational methods train and test models on data from the same cell line. However, the use of model trained from ChIP-Seq-tested cell lines to infer TFBS in experimentally unexplored cell lines is more useful in practice. In ENCODE-DREAM Challenge competition, such cross-cell TFBS prediction was proposed, in which J-team’s model won the championship by use of motif and DNase-seq (Keilwagen *et al.* 2019). Cross-biosample TFBS prediction requires epigenetic information, and therefore the architectures of DL models must be optimized to make them applicable to the integration of multiomics. Current transformer-based (Vaswani *et al.* 2017) models lack the ability to incorporate multiple types of biological information to the network. Although DNABERT has demonstrated impressive performance in TFBS prediction, its pretraining process is time-consuming and resource-intensive (Acheampong *et al.* 2021), making it unaffordable for most researchers.

To solve these problems, we propose TFTF, a novel multimodal DL architecture which utilizes multiomic information together with a modified network structure that integrates a “Balancer” layer. TFTF demonstrates highly accurate identification of TFBS within cell lines, as well as unexplored biosamples in cell type and species-specific way. Genome-wide scanning of TFBS illustrates the sensitivity of TFTF in TFBS. We further explain the underlying mechanism of TFTF model on both TFBS and non-TFBS regions. The multiomics used in this study (histone modifications or chromatin profiles) are significantly easier to be experimentally obtained than TFBS, making our method highly efficient, less cost and broadly applicable.

## 2 Materials and methods

### 2.1 Datasets

In this study, we utilized the sequencing data from reference genomes GRCh37 and mm10 for human and mouse, respectively. Liftover tool (Kent *et al.* 2002) was used to convert any data from other reference genome versions. We collected the DNA sequence and H3K4me3 signal (*P*-value column of BigWig file) from each cell line (Supplementary Fig. S1b), as well as H3K4me1 and DNase-seq signals from HSMMtube, NH-A, NHDF-Ad, Osteobl, IMR-90, Fibrobl, GM12878, H1 and HUVEC cells. Transfer learning is conducted with Bruce4 cell from mouse. The CTCF and EZH2 binding sites are obtained through ChIP-seq experiment, followed by IDR (Li *et al.* 2011) analysis according to the ENCODE protocol. We define the region of 101 bp flanking the center of TFBS peak as positive sample.

The union of all positive samples from various cell lines are treated as the positive dataset. To generate the negative data, TFBS peaks from the positive data were marked. For example, in the 50-cell-lines task, all the peaks from those 50 cell lines were marked. Similarly, in the seven cell lines task, the TF peak regions of the seven cell lines were marked. The negative DNA sequences are 101 bp long, randomly sampled from the human reference genome outside the marked regions, and at least 2 kb away from the boundary of the marked region. For each task, the same amount of negative samples were generated as those of the positive ones. For both positive and negative data, similar proportion of samples are outside the repetitive regions of human genome, which is also very close to the nonrepetitive-region ratio of GRCh37 (∼47%, Supplementary Fig. S2d). Hence, the construction of our experimental dataset simulates the similar distribution of human genome repeats in TFBSs. Furthermore, to calculate the multiomic information of the negative samples, we randomly selected a cell line from the positive dataset, and extracted the histone signal at the regions of negative peaks. For the seven cell lines task, we randomly sampled an amount of genome-wide negative peaks equal to the number of TFBS in the validation and testing cell lines, which were at least 2 kb away from positive peaks.

**Figure 1.**
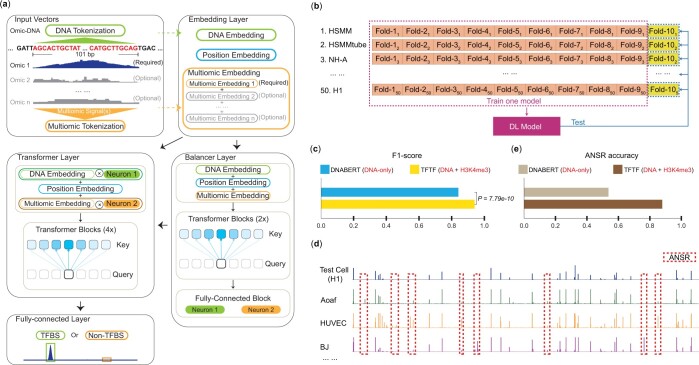
TFTF super accurately identifies TFBS in a 50-cell-line dataset. (a) The architecture of the TFTF model. The model takes both DNA sequence and multiomic information as input. It is composed of embedding, transformer, fully connected layers, together with a novel balancer layer to evaluate the weights of DNA and multiomic modification embeddings. In most cases we used DNA+H3K4me3 as input. TFTF is also capable of integrating the signals of multiple omics. (b) Ten-fold cross-validation process of the TFTF model on 50 cell lines. Positive samples (CTCF peaks) were randomly and equally divided into 10 folds. Corresponding negative samples with the same number of positive samples were generated for each fold. The union of nine folds of all 50 cells were used as the training data, and the remaining as the test data. This process was repeated 10 times. (c) Trained in union of 50 datasets, TFTF outperformed DNABERT by 9.97%. *P* value was calculated by Wilcoxon Signed-Rank Test. (d) Additional Negative Sampling Regions (ANSR) was designed to assess the model’s ability to distinguish TFBS across cell lines. Samples from ANSR are TFBS for other cell lines but non-TFBS in the test cell line. Regions from ANSR should be predicted as negative samples. (e) In ANSR, TFTF had 34.24% improvements over DNABERT.

In addition, we defined a unique negative dataset to be used for testing, named Additional Negative Sampling Regions (ANSR) (Fig. 1c). The regions in ANSR are the TFBS of other cell lines from the training dataset, but not the TFBS of the target from the testing dataset. Therefore, they are considered as negative dataset. The size of ANSR for each experiment is about $\frac{1}{5}$ the amount of the common testing data, and the test performance is evaluated separately.

### 2.2 Model architecture

The TFTF model consists of four components (Supplementary Fig. S1a): (i) an embedding layer incorporating both DNA sequence and multiomic information, (ii) a Balancer layer that calculates the weights between DNA sequence and multiomic signals, (iii) transformer blocks, and (iv) a classification layer for TFBS prediction.

#### 2.2.1 Embedding layer

The raw inputs of the TFTF consist of a 101-bp DNA sequence and the corresponding multiomic signal *P*-values at each base pair. We leverage the widely used k-mer representation to tokenize the DNA sequence (Chor *et al.* 2009), in which each nucleotide is concatenated with the preceding k-1 nucleotide. For example, sequence “ATCGTC” is transferred into “ATC, TCG, CGT, GTC” in 3-mer tokenization. The DNA vocabulary comprised all possible combinations of the k-mer plus three additional tokens: [CLS] to mark the sequence start, [SEP] to mark the sequence end, and [PAD] to pad the input. Consequently, the vocabulary size is $4^{k}+3$. In TFTF, *k* is set to 3, resulting in a vocabulary size of 67. Each DNA sequence begins with the [CLS] token, followed by the tokens of the DNA sequence, and ending with the tokens [SEP] and [PAD] to meet model input requirements.

For continuous multiomics information, we apply a categorical approach to generate 36-word tokens from the input signal value (Supplementary Fig. S1a). Therefore, different multiomic language word vectors can represent different signal intensity. The start, end, and padding are the same as the DNA sequence, such that a 39-integer token of multiomic signals forms a 39-word sized multiomic vocabulary.

With 3-mer tokenization, the DNA sequence length is reduced from 101 to 99. The central 99 values of the input multiomic signal will be used so that both parts of the inputs have the same length. Including the [CLS] and [SEP] tokens will result in a 101-word length for both the DNA and histone sequences before the zero padding.

For DNA sequence, sequence position and each of the multiomic signals, specific trainable embedding blocks are constructed. Given the vocabulary size in the embedding blocks, we assign dimension to eight, which is much smaller than the original DNABERT model (512) and thus greatly enhance the computing efficiency (Gu *et al.* 2021). The model input length is set to 128, and the size of all embeddings is 128×8.

#### 2.2.2 Balancer layer

In order to train dynamic weights for the outputs from both DNA sequence and multiomic embedding blocks, we have designed a novel module called “Balancer” layer. This layer takes the summation of the all embedding block as its input. Two transformer blocks with multi-head self-attention mechanisms followed by a feed-forward block are employed here in a manner similar to the encoder part of the original Transformers architecture (Vaswani *et al.* 2017). The dimension of the attention head’s weight matrix is reduced to 16. Suppose we have an initial embedding *E*, multi-head self-attention can be performed as:

(1) $$\mathrm{MultiHead}(E)=\mathrm{Concat}(\mathrm{hea}d_{1},\mathrm{hea}d_{2},\ldots ,\mathrm{hea}d_{8})\cdot W^{O},$$

where

(2) $$\mathrm{hea}d_{i}=\mathrm{softmax}(\frac{(EW_{i}^{Q})\cdot {\left(EW_{i}^{K}\right)}^{T}}{\sqrt{d_{k}}})\cdot EW_{i}^{V},$$

${\left[W_{i}^{Q},W_{i}^{K},W_{i}^{V}\right]}_{i=0}^{8}$ are trainable linear projections which convert the input dimension from 8 to 16, while the learned $W^{O}$ weight matrix projects the concatenated multiple heads into the shape of the input vector. The feed-forward unit in each transformer block consists of two fully connected layers, with residual connection strategy (He *et al.* 2016) and layer normalization being identical to the original paper.

As two transformer blocks capture the initial contextual information of the input embeddings, a fully connected block is added, learning from the output representations on how to balance the DNA and the sum of multiomic embeddings. We presented detailed network parameters in Supplementary Fig. S1a. The outputs of the two neurons will be activated by the following function, which will produce a DNA weight and a multiomic weight in the range of 0–2, where *x* is the output from either $\mathrm{neuro}n_{1}$ or $\mathrm{neuro}n_{2}$:

(3) $$\mathrm{Weight}(x)=1+\frac{e^{x}-e^{-x}}{e^{x}+e^{-x}},$$

The DNA and the sum of multiomic embeddings are multiplied by the DNA and multiomic weights respectively to optimize their scaling. We then calculate the sum of the re-scaled DNA and multiomic embeddings along with the position embedding, to form the final input embedding. During the training and predicting process, this balancer layer will dynamically generate DNA and multiomic weights.

#### 2.2.3 Transformer layer

The transformer layer is similar to the original transformer paper with a few modifications. We use four transformer blocks in the layer, and all the parameters of each transformer block are the same as the balancer layer.

#### 2.2.4 Down-stream TFBS prediction layer

The down-stream TFBS prediction layer is a simple fully connected network (detailed structures are shown in Supplementary Fig. S1a).

Our TFTF model has a total of only 32 320 parameters in the transformer layer, which promises extremely fast computation speed. This is significantly less than the DNABERT model, making it computationally efficient and resistant to over-fitting, which is advantageous for the small TFBS datasets.

### 2.3 Model training and evaluation

#### 2.3.1 Models trained for cross-validation

For the 50-cell-lines task, in order to compare to DNABERT, we used the parameters similar to those used in DNABERT-3, except for the input length which we set to 128 bp instead of 512 bp, as we focused our analysis on TFBS prediction, while DNABERT was used for multiple scenarios. The token length is set to 3 and the pretraining settings are the same as DNABERT. To optimize the 2-class classification of TFBS, Adam optimizer (Kingma and Ba 2015) with a learning rate of 0.0001 was used in conjunction with a cross-entropy loss:

(4) $$\mathrm{Loss}(x,y)=-\frac{1}{N}\sum_{n=1}^{N}log\left(\frac{\;\text{exp}(x_{y_{n},n})}{\;\text{exp}(x_{1,n})+\text{exp}(x_{2,n})}\right),$$

where $y_{n}\in \{1,2\}$ is the ground truth labels, $x_{1}$ and $x_{2}$ are the outputs of the very last fully connected layer, N is the batch size.

For the 10-fold cross-validation, we randomly divide the dataset of each cell line into 10 equal folds. In each time, nine folds are used for training and the remaining one for testing. This process is repeated 10 times. The training process involves the union of training folds of all 50 cells with the parameters randomly initialized and kept the same every time. Models are trained until the loss value of the last 50 consecutive batches decreases <0.01 compared to the current batch, using a batch size of 2500 on an NVIDIA P100 GPU. All the learning hyper-parameters are determined using a grid search algorithm.

#### 2.3.2 Models trained for TFBS prediction in unexplored cell lines

For the unexplored cell prediction task, the union TFBS peaks in the training cell lines will be used as the positive data. Adam optimizer and cross-entropy loss were employed for both TFTF and DNABERT. DNABERT was trained with a batch size of 4000 and a learning rate of 0.0001, while the TFTF model utilized a batch size of 2500 and the same learning rate. Performance of validation dataset was evaluated after each epoch, with early stop implemented when the validation results converged (F1-score of the following four consecutive epochs increased <0.2% compared with current epoch). All models were trained in NVIDIA P100 GPU.

#### 2.3.3 Data augmentation

Given a TFBS ChIP-seq peak spanning from the *a*th nucleotide to the *b*th nucleotide on its chromosome, the corresponding original positive dataset’s start location can be calculated as $\mathrm{Star}t_{\mathrm{org}}=\frac{a+b}{2}-50$. The start locations of the 14 augmented data can be expressed as follows:

(5) $$\mathrm{Star}t_{\mathrm{aug}}=\frac{a+b}{2}-50\pm i,(i=1,2,3,\ldots ,7),$$

The end location is $End_{\mathrm{org}}=\mathrm{Star}t_{\mathrm{aug}}+101$. To generate a sufficient amount of negative data, we sampled 14× more negative samples. Therefore, the augmented dataset derived from seven cell lines should be 15 times the size of the original dataset. As a result, the new dataset consists of nearly 10 million samples.

#### 2.3.4 Evaluation metrics

In this study, the amount of positive data size was equivalent to the amount of negative data size. We utilized precision, recall, and F1-score to evaluate the model’s performance: $\mathrm{Precision}=\frac{TP}{TP+FP}$, $\mathrm{Recall}=\frac{TP}{TP+FN}$, $F1=\frac{2P\mathrm{recision}\times \mathrm{Recall}}{\mathrm{Precision}+\mathrm{Recall}}$, where TP, TN, FP, FN stands for true positive, true negative, false positive, and false negative, respectively. For genome-wide TFBS scanning, we used area under precision–recall curve and receiver operating characteristic curve (auPRC and auROC) to evaluate the outcome for heavily imbalanced samples.

For ANSR analysis, suppose *N* is the total number of ANSR samples, *n* of them are predicted as negative; The ANSR accuracy is defined as:

(6) $$\mathrm{ANSR}\;\;\mathrm{Accuracy}=\frac{n}{N},$$

During the training, to minimize the loss function, for positive samples, $x_{1}-x_{2}$ should be enlarged, and vice versa for negatives. Therefore, a greater $x_{1}-x_{2}$ indicates a higher confidence that the sample shall be predicted as positive in machine learning. We defined the Confidence Score of TFTF prediction as the difference between the two output values in the downstream classifier at the very end of TFTF model:

(7) $$\mathrm{Confidence}\;\;\mathrm{Score}=x_{1}-x_{2},$$

To evaluate the attention of the TFTF and DNABERT models in different regions, we define the attention score for the $j^{th}$ token as:

(8) $$\mathrm{Attention}\;\;\mathrm{Score}(j)=\sum_{i=1}^{L}So\mathrm{ftmax}(\frac{q_{i}\cdot k_{j}}{\sqrt{d_{k}}}),$$

where $q_{i}$ and $k_{j}$ are the *i*th and the *j*th vectors in Queries and Keys, respectively. *L* is the model input length.

We defined the following equation for fold change of attention score (ASFC), to quantify the model attention inside and outside motif regions:

(9) $$\mathrm{ASFC}=\frac{1}{8}\sum_{h=1}^{8}\frac{\sum_{j\subseteq M}\sum_{i=1}^{L}So\mathrm{ftmax}(\frac{q_{h,i}\cdot k_{h,j}}{\sqrt{d_{h,k}}})}{\sum_{j⊈M}\sum_{i=1}^{L}So\mathrm{ftmax}(\frac{q_{h,i}\cdot k_{h,j}}{\sqrt{d_{h,k}}})},$$

where h indicates the *h*th head, *M* stands for the TFBS motif region, and the mean value of the eight heads of attention score is used to calculate ASFC for each sample.

### 2.4 Motif analysis

We used FIMO (Version 5.5.0, https://meme-suite.org/meme/tools/fimo) to scan for targeted motifs in peak region (Grant *et al.* 2011), and used its result to determine whether the motif exists and its location. The *P*-value was set to .001. We applied MEME (Version 5.5.0, https://meme-suite.org/meme/tools/meme) in classic mode with default parameters to discover de-novo motifs (Bailey *et al.* 2009). We employed TomTom (Gupta *et al.* 2007) (Version 5.5.0, https://meme-suite.org/meme/tools/tomtom) to map the de-novo motifs to known motif databases, selecting “eukaryote DNA, vertebrates (*in vivo* and *in silico*)” as the motif database and Pearson correlation coefficient as the index. The matched motifs with the smallest *P*-value were selected.

### 2.5 ChIP-qPCR experiment

The predicted CTCF binding regions were used to design qPCR primer using NCBI Primer-BLAST. hESC H1 cells were collected in QuickExtract DNA Extraction Soln and were then digested as CTCF qPCR control. CTCF enrichment of the predicted regions were normalized to three CTCF nonbinding regions on the genome, respectively. We performed three technical replicates for each sample.

### 2.6 Statistics

Wilcoxon Signed-Rank Test (Rey and Neuhäuser 2011) was used to evaluate the difference of F1-score. Fisher’s *z* Transformation (Welz *et al.* 2021) was used to compare Pearson correlation coefficients. Wilcoxon Signed-Rank Test was used to compare the ASFC values. Student *t*-test was used to compare ChIP-qPCR results.

## 3 Results

### 3.1 TFTF super accurately identifies TFBS in a 50-cell-line dataset

The TFTF model consists of four parts, embedding layer, balancer layer, transformer layers and fully connected layer (Fig. 1a, Supplementary Fig. S1a). To improve upon the traditional transformer networks (Vaswani *et al.* 2017), we used both DNA sequence and multiomic information in the embedding layer, instead of DNA sequence only used by DNABERT (Ji *et al.* 2021). TFTF is flexible with using different combinations of multiomic signals as input. However, in this work, unless otherwise specified, we used *P*-value of H3K4me3 ChIP-seq signal over control as the main multiomic information by default. H3K4me3 was chosen as it is the most experimented histone modification in the ENCODE project (The ENCODE Project Consortium 2012), and it is related to TFBS (Robertson *et al.* 2008). Besides the increased accuracy, the multiomic information is also useful for cell type-specific prediction. In addition, a novel “Balancer” layer was added to the model immediately following the embedding layer. This layer allows for the weights of DNA sequence and multiomic embedding vectors to be trained and automatically calculated (Section 2).

In this section, we collected CTCF (Ong and Corces 2014) peaks calculated by irreproducible discovery rate (IDR) (Li *et al.* 2011) in 50 cell lines (Supplementary Table S1) from ENCODE project, served as the positive dataset. For the negative dataset, we randomly sampled regions (same peak size as the positive samples, Section 2) 2 kb away from CTCF peaks of all 50 cells, irrespective of their GC-content. The number of negative regions was equal to that of positive regions for every cell. A modified 10-fold cross-validation was used for evaluation (Fig. 1b). For every cell line, the union of positive and negative datasets were randomly separated into 10 folds (groups), where the number of positive and negative regions was kept the same. A trial run was performed in which the first nine folds of all 50 cells served as training data and the remaining one fold of all 50 cells was left for testing. This process was repeated 10 times for each fold left for testing and the mean value of the 10 processes was taken as the final result. TFTF showed 5.66%–14.35% improvement for each cell line (Supplementary Fig. S1b), and achieved an impressive F1-score of 94.05% (Section 2). This marked a 9.97% increase in overall performance when compared to DNABERT ($P=7.79\times 10^{-10}$) for all 50 cells combined (Fig. 1c).

We then defined a special group of TF negative regions (Fig. 1d), which represent TFBS (positive) in other cells but non-TFBS (negative) in the target cell line. We named these regions Additional Negative Sampling Regions (ANSR, Section 2). Using TFTF to predict ANSR, the mean value of 10-fold cross-validation was used as the final result. This showed impressive 87.86% accuracy, outperforming DNABERT by 34.24% (Fig. 1e). The result implied that DNA-based methods such as DNABERT are unable to distinguish genomic regions with different TFBS among cell lines. In contrast, TFTF had a strong ability to learn across multiple datasets and identify cell type-specific TFBS—a capability that is highly desirable in real-world applications.

To explore the contribution of the balancer layer to TFTF, an ablation study was performed with or without this layer. The results showed that TFTF with a balancer layer had 94.05% in F1-score and 87.86% in accuracy of ANSR, with improvements of 3.63% ($P=7.79\times 10^{-10}$) and 5.68% compared to TFTF without the balancer layer (Supplementary Fig. S1c). This suggested that the input weights between DNA sequence and histone modification are not the same for all regions, with DNA sequence being more important for some and histone modification for others.

### 3.2 TFTF is able to predict TFBS in experimentally unexplored biosamples

**Figure 2.**
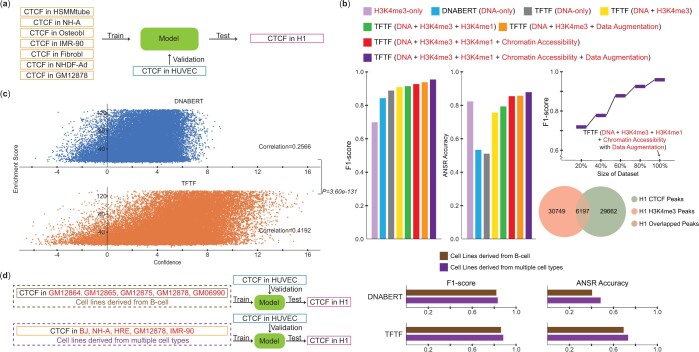
TFTF is able to predict TFBS in experimentally unexplored biosamples. (a) Illustration of seven cell lines used to train TFTF model, then validated in HUVEC cell and finally tested in H1 cell. (b) Performance comparison of models trained on DNA+histone, DNA only, histone only, additional histone modification information, additional chromatin profile information, and data augmentation process. Here we also presented TFTF trained on DNA+histone, but made predictions based on DNA only. (c) Correlation between ChIP-seq signal intensity and Confidence Score showed 16.26% better correlation of TFTF compared with DNABERT. *P* value was computed by Fisher Z-Transformation. (d) Comparison of TFTF model trained by B-cell derived cell lines and multiple cell types derived cell lines in terms of F1-score and accuracy of ANSR for both models.

To replicate real-world applications of identifying TFBS in TF-ChIP-seq-untested cell lines, we trained the TFTF model with CTCF binding sites in seven cell lines (HSMMtube, NH-A, NHDF-Ad, Osteobl, IMR-90, Fibrobl, and GM12878). We then tested the performance in an unexplored cell line H1 (Fig. 2a), and included an independent validation set with CTCF TFBS from HUVEC cell. The training process will be stopped when the validated F1-score in HUVEC cell converged (Supplementary Fig. S2a), and the trained model will be utilized to assess the H1 dataset. The results showed that TFTF had 90.97% in F1-score and 75.68% in ANSR accuracy, with improvements of 6.68% and 22.35% compared to DNABERT (Fig. 2b). To contrast the results, a model with only histone modification as the input was tested, and the F1-score fell 21.07% short of the combined DNA sequence and histone modification input. Nonetheless, this score was still much better than using histone peak as the TFBS indicator (only 17.28% overlapped peaks, Fig. 2b).

Here we check with the impact of using more multiomic information in the model. We first introduced another histone modification information, H3K4me1 (Robertson *et al.* 2008), to TFTF. The additional histone information improved the performance of the TFTF model by 0.55% and 4.70% for F1-score and ANSR, respectively (Fig. 2b). This suggests that including more histone modification information in the TFTF model is beneficial. In addition, we incorporated DNase-seq signals that profile the chromatin accessibility into TFTF, which could further improve the F1 and ANSR by 1.4% and 4.95% (Fig. 2b). TFTF is therefore able to integrate diverse multiomics data toward even better performance.

It’s interesting to see TFTF performance if no epigenetic information is available in test data. We still used DNA+H3K4me3 as input to train TFTF, while made predictions solely based on DNA sequence by manually freezing the Balancer weights as 1 and 0, for DNA and histone, respectively. With DNA-only input, TFTF yielded 88.79% in F1-score (Fig. 2b), outperformed DNABERT by 4.5%, which is only 2.18% less than using the complete DNA+H3K4me3 information to do the prediction. Therefore, it is useful to train TFTF in biosamples with fully experimented multiomics, and make predictions in biosamples that lack epigenetic markers. However, multiomic information is important to maintain good cell type-specific results, as TFTF’s ANSR dropped to 50.92% without epigenetic information.

It is widely accepted that increasing the dataset size enhances performance of the model, particularly in situations with limited datasets (Wong *et al.* 2016). To address this issue, we implemented a data augmentation method of shifting TF peak regions within a range of ±7-bp from the peak center, forming extra 14× more positive dataset for training (Section 2, Supplementary Fig. S2b), and creating 14× more negative dataset randomly sampled from the regions outside the union peaks of the seven cell lines. It is reasonable to assume that shifting the peaks is beneficial as TF motifs are not located exactly in the center of the peak region. By utilizing this method, the F1-score for TFTF was improved to 93.86% (Fig. 2b), and the accuracy of predicting ANSR was increased to 85.78%.

By incorporating all multi-omics information and data augmentation mentioned above, we observed TFTF had 95.28% in F1-score and 87.76% in ANSR accuracy, with significant improvements of 11.0% and 34.43% compared to DNABERT. To investigate the impact of dataset size on the model, we randomly sampled 20%, 40%, 60%, and 80% and 100% of the integrated dataset and applied it as input to the TFTF model. The results showed a positive correlation between F1-score and the size of the dataset (Fig. 2b).

We then examined the correlation between the Confidence Score (Section 2) generated by TFTF prediction output, and the ChIP-seq signal intensity of positive samples in H1. Higher Confidence Score indicates that TFTF is more likely to predict the sample as positive. As anticipated, TFBS with higher ChIP-seq experiment enrichment scores had a positive correlation to the Confidence Score, with a correlation coefficient of 0.4192 (Fig. 2c); conversely, the correlation coefficient for DNABERT was only 0.2566 (*P* = 3.60 × 10^−131^).

The selection of more diverse data for training ML models improves generalization and accuracy. Here we used two groups of training dataset for comparison. One group comprises GM12864, GM12865, GM12875, GM12878, and GM06990, which are derived from the B-cell. The other group includes BJ, NH-A, HRE, GM12875, and IMR-90, which are derived from multiple cell types (Fig. 2d). The total number of peaks between two groups are comparable, 210 484 versus 211 098 (Supplementary Fig. S2c). Negative samples were generated accordingly, similar to the above procedures. For simplicity, we only used H3K4me3 as the histone information, and without data augmentation. The results showed a 2.01% performance improvement when using the multiple cell types derived cell lines, compared to the B-cell derived cell lines (Fig. 2d). This indicates that it’s benificial to build TFTF models using more diverse cell types.

Overall, this result suggests that TFTF is highly accurate in predicting TFBS in unexplored biosamples, outperforming current SOTA methods. Moreover, including more histone modification information, processing with data augmentation, increasing the size of dataset, and selecting the dataset from multiple cell type derived cells are critical for making the model robust and precise.

### 3.3 TFTF enables robust genome-wide detection for TFBS in unexplored biosamples

**Figure 3.**
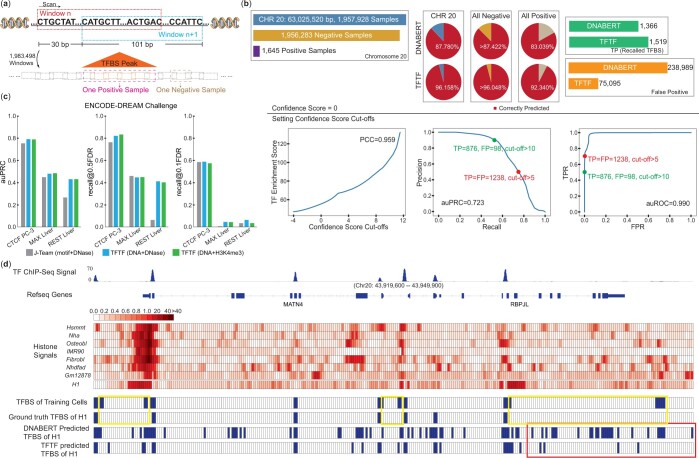
TFTF enables robust genome-wide detection for TFBS in unexplored biosamples. (a) Genome-wide scanning of CTCF TFBS from H1, using a 101-bp windowed bin size and a 30-bp sliding window size. Chromosome 20 was used for demonstration. (b) At 0 confidence score, TFTF recalled 9.30% more peaks and reduced nearly two thirds of FP calls compared with DNABERT. By setting different Confidence Score cut-offs, we observed 0.723 auPRC and 0.990 auROC. The red and green dots mark 0.753 and 0.533 recall of ChIP-seq peaks at 0.5 and 0.1 FDR. (c) Comparison between J-team’s model and TFTF in prediction of three kinds of cross-cell TFBS in the ENCODE-DREAM Challenge datasets. (d) A 30-kb region around MATN4 and RBPJL genes in H1 cell. CTCF ChIP signal from H1 and H3K4me3 ChIP signal from seven training cells plus H1 are shown in blue and red, respectively. Four types of TFBS regions are marked in blue in the last four rows, with the H1 ground truth showed inconsistent TFBS between the seven training cell lines and H1. DNABERT made frequent FP predictions, while TFTF was able to differentiate inconsistent TFBS, largely reducing FP predictions.

Practical TFBS discovery is quite different from predicting with prepared candidate regions, which are often unavailable. Here we applied the TFTF model with data augmentation trained by DNA+H3K4me3 in the seven distinct cell lines to scan the genome-wide CTCF TFBS in H1 cell. To save computational resources and without losing generality, we scanned the genome-wide TFBS on chromosome 20 only. The length of the scanning window was set to 101 bp, and the sliding window was 30 bp (Fig. 3a). All windows that overlapped with the same TFBS peak were considered one positive sample, and if positive prediction was made in any of those windows, true positive (TP) prediction was calculated only once. All windows outside peak regions were considered negative samples. A total of 1 957 928 regions were generated, including 1645 positive and 1 956 283 negative. By default (confidence score = 0), TFTF recalled 1519 TFBS, which was 92.34% of the total 1645 TFBS on chromosome 20, 9.30% more than DNABERT. In addition, TFTF achieved an 8.63% improvement than DNABERT in negative regions (Fig. 3b). Note both models showed improved performance in negative regions compared to the results of previous sections. It’s due to the larger number of genome-wide negative regions compared to the number of ChIP-seq peaks. Although there were many false positive (FP) predictions, TFTF successfully reduced the number of FP from 238 989 to 75 095 whilst still being able to successfully identify 9.30% more true TFBS when scanning the entire chromosome (Fig. 3b).

The aforementioned positive correlation between TFTF Confidence Score and TF ChIP-seq enrichment provided an approach for prioritization of the most trustworthy predicted peaks by setting Confidence Score cut-offs. When gradually increasing the Confidence Score cut-offs, peaks that passed the thresholds exhibited linear improvements of average CTCF enrichment score with stronger TF binding tendency (Fig. 3b, with 0.959 correlation coefficient). Therefore, it is possible to filter the massive false positives based on Confidence Score. In the genome-wide scanning of chr20, through changing Confidence Score thresholds, we evaluated TFTF performance by precision–recall curve (PRC) and receiver operating characteristic (ROC) curve. We observed good area under PRC (auPRC) of 0.723, as well as an impressive area under the ROC curve (auROC) of 0.990 (Fig. 3b). We marked two useful cut-off values: (1) Confidence Score>5 (red dot), which recalled 1238 true TFBS (75.26%), while decreased the FP number to 1238 (0.5 precision); and (2) Confidence Score>10 (green dot), which increased the precision to 0.9 (only 98 FP), while maintained 53.25% recall (876) of ChIP-seq peaks.

We further examined TFTF’s ability for cross-cell TFBS detection by applying TFTF to ENCODE-DREAM Challenge dataset, which also simulates the heavily imbalanced classifications in genome-wide scanning. Taking in the same DNA+DNase-seq input as J-team’s model (ENCODE-DREAM Challenge champion), TFTF yielded 3.74%, 3.07%, and 16.48% improvements of auPRC in prediction of cross-cell CTCF, MAX, and REST, respectively. Besides, in most cases, TFTF can recall more ChIP-seq peaks at certain false discovery rate (FDR, 1-precision) cut-offs (e.g. when 0.5 FDR is required, TFTF can recall 35.18% more true TFBS, compared with J-team’s model in prediction of REST in liver). TFTF exhibited similar performance when changing to use DNA+H3K4me3 as input. Therefore, TFTF can super accurately detect various genome-wide TFBS in unexplored biosamples.

With an increase of performance of TFTF over DNABERT ranging from 8.63%–9.30%, we sought to investigate the potential reasons. Taking a 30-kb track (43 919 600–43 949 900) of chromosome 20 as an example, we presented the histone ChIP-seq signals from both training and testing cells, in which higher signal intensity was reflected by darker red color (Fig. 3c). We also marked the TFBS in blue color. In the first two tracks of TFBS, there are many inconsistencies in the TFBS between the training and testing datasets at the same genomic locations (yellow box). These cell type-specific TFBS cannot be distinguished by the DNA-only model since the DNA sequences are identical. Moreover, the last part (red box) of this track in the training cells are non-TFBS in H1, and DNABERT made frequent FP predictions (Fig. 3c). In comparison, TFTF was able to reduce the number of FP and distinguish the inconsistent TFBS in the same location. It is clear that the DNA-sequence alone is not enough to accurately predict TFBS in different cell lines of the same species and can lead to results similar to that of ANSR prediction (Fig. 2b). Thus, TFTF was able to identify cell type-specific TFBS together with high precision.

### 3.4 TFTF consistently identifies true TFBS in threshold tuning-free way

Replicates are frequently used in biological experiments to reduce uncertainty and increase reproducibility (Landt *et al.* 2012). The IDR threshold method (Li *et al.* 2011) is widely used to identify TFBS regions which are consistent between replicates. However, regions with less enriched and inconsistent signals between replicates are difficult to be dealt with, and are typically discarded by IDR. Peak calling program (Feng *et al.* 2012) or the IDR threshold method usually demand manual adjustment of parameters such as *P*-value, *q*-value and so on; Changes in such parameters can lead to varied results. The TFTF model, however, offers manually free way from parameters tuning, and yields stable results automatically calculated by the model.

**Figure 4.**
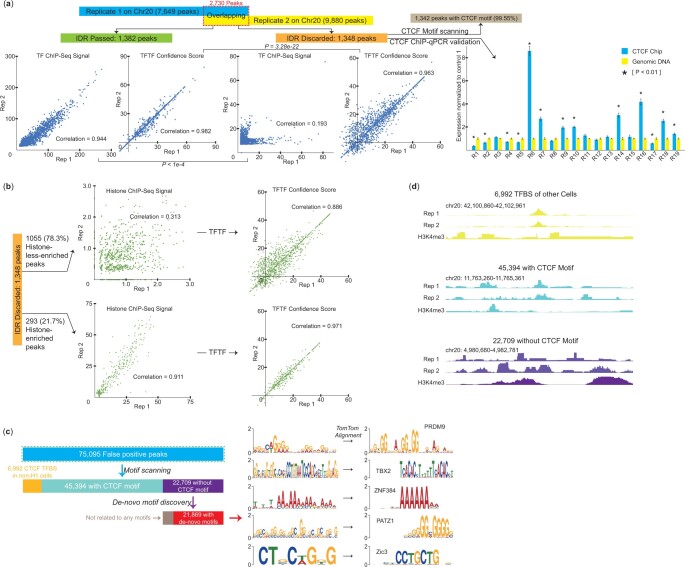
TFTF consistently identifies true TFBS in threshold tuning-free way. (a) CTCF ChIP-qPCR experiments were conducted in 19 regions randomly selected from IDR discarded peaks. The expression level of each region was normalized to the first control region (genomic background without CTCF ChIP signal). Motif was scanned by FIMO. The consistency of two replicates for 1348 IDR-discarded and 1382 IDR-passed CTCF TFBS peaks on chromosome 20 of H1 cell were evaluated by the ChIP-seq signal or Confidence Score. The correlation coefficients of ChIP-seq signals reduced from 0.944 to 0.193 between the two groups, while the correlation coefficients of Confidence Score remained close and high. *P* values were calculated by Fisher Z-Transformation. (b) The consistency of the replicates of histone input, and the TFTF predictions. (c) Scanning CTCF TFBS on chromosome 20 from H1, where 75 095 FP regions were predicted. Of these, 6992 were CTCF TFBS in non-H1 cells, 45 394 contained CTCF motif and 21 869 contained de-novo motifs. (d) For every type of FP regions, examples of CTCF ChIP-seq signals in FP regions of high Confidence Score for both replicates were shown.

We investigated how the TFTF model works with replicates by comparing it with IDR results, focusing on CTCF peaks on chromosome 20 of H1 cell. MACS peak caller program (Zhang *et al.* 2008) identified 7649 and 9880 CTCF peaks from two biological replicates, respectively. Of the 2730 shared peaks between the replicates, 1382 (IDR selected group) eventually passed the IDR threshold, while the rest 1348 (IDR declined group) were discarded. This is also reflected by the consistency of TF ChIP-seq signals between replicates (Fig. 4a), as dramatic difference (.944 versus .193, $P<1\times 10^{-4}$) of correlation coefficients can be observed between the IDR passed and declined groups. From those ChIP-seq-discarded peaks, we randomly selected 19 of them to perform CTCF ChIP-qPCR (Asp 2018) (Section 2, Supplementary Table S2), of which eight (42.11%) were found to be positive (Fig. 4a, Supplementary Fig. S3c). Besides, motif scanning suggested 99.55% of those 1348 discarded peaks contain CTCF motifs. Hence, the enriched TF signals in at least one replicate, the ChIP-qPCR experimental validation, as well as the *in silico* motif analysis, strongly indicated that those IDR-discarded regions are in fact extremely possible to be true TFBS, and it is likely that they cannot pass the IDR threshold simply because we cannot maintain good consistency in two times of biological experiments, resulting in a considerable amount of false negatives.

We further examined TFTF predictions on biological replicates by use of different histone replicates. As shown in Fig. 4b, nearly 78.3% of those IDR discarded peaks are less-enriched with histone signals. The 0.313 correlation coefficients between replicates of those peaks suggests that the histone experiments are also largely inconsistent, which provides different inputs for TFTF model. However, the output confidence scores from TFTF are very consistent with correlation coefficients of 0.886. For the 293 histone-enriched peaks, the correlation can go to 0.971. In total, for both IDR-passed and IDR-discarded groups, TFTF’s correlation coefficients between replicates were close and with high values (0.982 versus 0.963, $P=3.28\times 10^{-22}$, Fig. 4a). Besides, all ChIP-qPCR validated (but IDR declined) peaks were predicted as positive by TFTF. Consequently, TFTF can deal with the noise and variations of biological experiments, and yield much more consistent predictions among replicates, compared with TF ChIP-seq.

In addition, In the genome-wide scan of CTCF sites in chromosome 20 of H1 cell, at 0 confidence score, 75 095 FP peaks were identified by TFTF. We further investigated these regions to determine if they were mislabeled by peak calling methods or if any underlying biological mechanisms were responsible. 6992 of 75 095 peaks were CTCF TFBS in other cell lines. For the remaining 68 103 peaks, 45 394 contained CTCF motif (Grant *et al.* 2011) (Section 2). For the 22 709 peaks without CTCF motif, we performed de-novo motif discovery (Bailey *et al.* 2009) (Section 2) and identified the top five motifs which, when compared to known motif database (Gupta *et al.* 2007, Mathelier *et al.* 2014) (Section 2), detected PRDM9, TBX2, ZNF384, PATZ1 and Zic3 as the co-factors (Supplementary Fig. S3). 18 757 of 22 709 peaks exhibited one of these motifs (Fig. 4b), suggesting a shared regulation pattern. Furthermore, for each of the three groups (CTCF TFBS in other cell line, peaks with CTCF motif, peaks without CTCF motif), top 10 peaks with the highest Confidence Score scores were selected and the ChIP-seq signals of both CTCF and H3K4me3 (in bigWig format) were browsed (Fig. 4c, Supplementary Fig. S3b). Most peaks showed enriched signals, suggesting that they were potential TFBS.

### 3.5 TFTF attends to distinct parts of peak region

**Figure 5.**
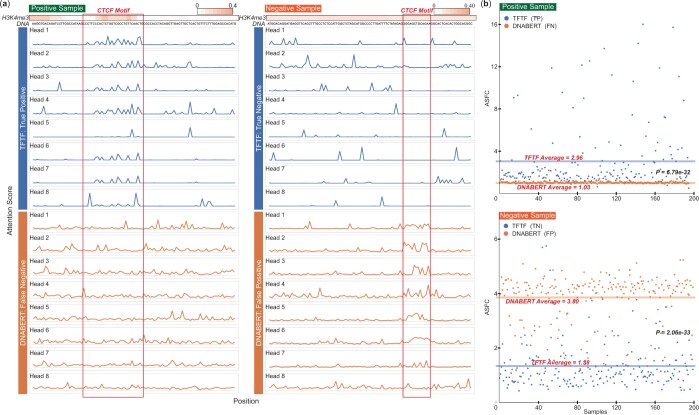
TFTF attends to distinct parts of peak region. Attention score of different types of samples at the peak regions were plotted. (a) A typical positive and negative sample. CTCF motifs were scanned and determined through the peak region. TFTF attended to the CTCF motif region in six out of eight heads for the positive sample, and had little attention in all eight heads for the negative sample. DNABERT, in contrast, had little attention to the CTCF motif region in almost all eight heads for the positive sample, and had enriched attention in seven out of eight heads for the negative sample. (b) By scanning CTCF motif on chromosome 20 of H1, we calculated attention scores for all samples with CTCF motifs. Fold change of attention score (ASFC) between inside and outside of CTCF motif regions was plotted in scattered dot. The mean values of ASFCs were calculated and plotted in bold line. All positive and negative samples where TFTF predicted correctly and DNABERT predicted wrongly were illustrated. TFTF showed more attention within the CTCF motif regions for positive samples, and general attention across peak regions for negative samples. In contrast, DNABERT showed the opposite results. The Wilcoxon Signed-Rank Test was applied to calculate *P* values.

The architecture of both TFTF and DNABERT include attention-based components which allow the model to focus on specific parts of the input. We quantified the amount of attention of each position by calculating its attention score (Section 2), plotting the results in curves for both positive and negative CTCF peak regions (Fig. 5a). As an example, we selected a positive and a negative CTCF TFBS, and scanned the CTCF motif in each peak region (Grant *et al.* 2011) (Section 2). For the positive CTCF peak, the attention scores (Section 2) were enriched in the CTCF motifs in six of eight heads of the TFTF model, but were less enriched in almost all eight heads of the DNABERT model (Fig. 5a, left part). For the negative CTCF peak, little attention scores were observed in CTCF motifs for all eight heads of the TFTF model, whereas seven out of eight heads were enriched in the DNABERT model (Fig. 5a, right part). These results demonstrate how TFTF attended to positive and negative peaks compared to DNABERT.

We investigated the fold change of attention score (ASFC) between inside and outside of CTCF regions of candidate CTCF peak in chromosome 20 to get the overview difference of attended regions between positive and negative samples (Section 2). We investigated ASFC in samples oppositely predicted by TFTF and DNABERT (Fig. 5b, Supplementary Fig. S4a). For positive samples, the results showed 2.87× enrichment ($P=6.79\times 10^{-32}$) of TFTF ASFC in TFTF correctly predicted samples, compared to only 1.64× enrichment ($P=3.12\times 10^{-8}$) of DNABERT ASFC in DNABERT correctly predicted samples. For negative samples, the results presented 2.75× enrichment ($P=2.06\times 10^{-33}$) of DNABERT ASFC in DNABERT wrongly predicted samples, compared to only 1.83× enrichment ($P=1.51\times 10^{-26}$) of TFTF ASFC in TFTF wrongly predicted samples. Notably, when both models were correctly predicted for positive samples, the averaged ASFC was 3.13× higher in TFTF model compared to DNABERT (Supplementary Fig. S4b, $P<2.18\times 10^{-139}$). These results suggest that with the help of histone modification information, TFTF was more effective than DNABERT in identifying CTCF TFBS, as it was capable of recognizing positive samples by greater attending to the CTCF motif regions, while for negative samples it gave attention to the entire peak regions.

### 3.6 TFTF can be reliably generalized to distinct species, TFs, and abnormal cells

**Figure 6.**
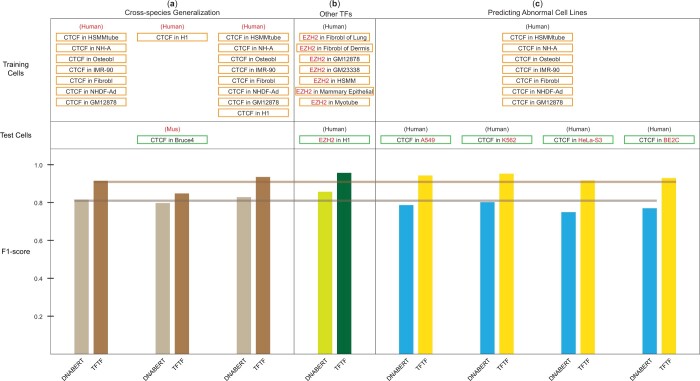
TFTF can be reliably generalized to distinct species, TFs and abnormal cells. (a) We trained three different TFTF models using seven human cells (left), H1 cell only (middle), and seven cells + H1 cell (right) and tested on mouse Bruce4 cell. TFTF showed strong generalization ability across species than DNABERT. (b) TFTF model was used for prediction of EZH2 TFBS. The same H3K4me3 information was used as the prediction of CTCF TFBS. Seven human cells were used for training and H1 for testing. TFTF was shown to have 9.99% improvements compared to DNABERT, indicating good generalization of TFTF to multiple TFs predictions. (c) TFTF was trained using seven human cells (normal) and was tested on four abnormal cell lines: A549, K562, Hela-S3, and BE2C. Results showed that TFTF had a significant improvement in accuracy compared to DNABERT.

It is useful to train TFTF model in *Homo sapien* and then transfer the model to other species for which experimental datasets are lacking. We tested the generalization of the model trained by seven human cell lines +/- H1 cell on the mouse Bruce4 cell, which has a similar stage as human H1 cell. The model achieved 91.40% accuracy (Fig. 6a, left part), outperforming DNABERT by 9.65%. This implies that TFTF is able to generalize the learned DNA-histone patterns to other species without fine-tuning in the target species.

We then compared the results generated by the model trained using three datasets (7 cell lines without H1, H1 cell only, seven cell lines plus H1 cell). The data showed that F1-score is positively correlated with the number of cells (Fig. 6a). Even though H1 cell is more closely related to Bruce4 cell, the training set of seven cells without H1 still yielded a higher F1-score than H1 only.

We then investigated the generalization of TFTF to other TFs. With EZH2 as an example, we trained the model using the EZH2 ChIP-seq peaks (called by IDR threshold method) from fibroblast of lung, fibroblast of dermis, GM12878, GM23338, HSMM, mammary epithelial, and myotube, and validated it on HUVEC with a test performance on H1. TFTF demonstrated a 9.99% improvement of F1-score compared to DNABERT (Fig. 6b). Notably, since histone modification information is the same for different TFs, TFTF can accurately predict all TFs in a cell from a single histone modification experiment.

Predicting TFBS in abnormal cell lines (e.g. tumor, cancer) is challenging due to the large genetic and epigenetic changes in these cells. To investigate whether histone modification can aid in this task, we trained TFTF and DNABERT models using data from seven normal cell lines, and tested them on data from A549, K562, HeLa-S3, and BE2C cells. Results showed that the performance of the DNABERT model decreased to an average F1-score of 77.57%, while the TFTF model achieved an impressive average F1-score of 93.40% (Fig. 6c).

## 4 Discussion

Hundreds of TFs have distinct cell type and species-specific binding sites, mandating millions of experiments to detect TFBS. However, existing experiments such as ChIP-seq are not consistent and sensitive enough to accurately detect all TFBS. To address this, we have developed a novel and cutting-edge transformer-based DL model TFTF which incorporates multiomics information to predict both in-cell and cross-cell TFBS with enhanced accuracy. In an in-cell prediction of 50-cell-lines dataset, TFTF achieved 94.05% in F1-score, with 9.97% improvement compared to the current SOTA DNABERT. In a real application of predicting TFBS in an experimentally unexplored cell, TFTF demonstrated 90.97% in F1-score, a significant improvement of 6.68% in F1-score, compared to DNABERT. Moreover, TFTF outperformed DNABERT (34.24% and 22.35% for in-cell and cross-cell tasks) on a special negative dataset ANSR, which is hard to be distinguished across cells based on DNA sequence alone. The useful balancer layer of TFTF enables the model to integrate and assign weights to the input of both DNA sequences and histone modification data, which allowed for a 3.63% lift in F1-score. More types of histone modification and chromatin profile datasets, data augmentation and multiple cell types derived cells can lead to an optimized TFTF model with improved F1-score of 95.28%. In addition, in genome-wide scanning for across-cell TFBS, TFTF outperformed the ENCODE-DREAM Challenge champion in prediction of multiple TFs. Furthermore, 42.11% of the FP predicted TFBS were verified as TP by ChIP-qPCR experiment, highlighting the model’s reliability and accuracy compared to peak calling methods.

It is reasonable to include histone modification or chromatin profile information in TFTF, as it is typically easier to be obtained than the binding sites of many TFs which rely on the quality of the antibody used. With its advantages of providing information across cells, the multiomic data contributes additional information to the TFTF model to make cell type-specific and cross-species prediction available. Moreover, TFTF model can be expanded to additional TFs as well as abnormal cells. This is the first to exhibit the capability of identifying cell type-specific and species-specific TFBS, which cannot be accomplished by DNA sequence-based models.

To further explain the efficiency of TFTF, the ASFC was used to detect the difference between regions within and outside of the canonical motif of the targeted TF. For the positive samples, TFTF focused mainly on the canonical motif region of the targeted TF, resulting in a high ASFC. While for the negative samples, TFTF gave attention to the entire peak regions. This suggests that TFTF may provide different rules for different samples.

The model can be further improved by considering multiple histone modifications such as H3K27ac and H3K4me1, as well as other epigenetic markers such as DNA methylation and DNA hypersensitivity. Due to the varying nature of epigenetic information across cell lines, it is challenging to pretrain to effectively utilize both DNA sequence and multiomic data. Thus, it is important to explore more efficient and reasonable pretraining methods in order to facilitate easier applications and expansion of the model. In addition, larger, more complex model is useful and could be developed with more datasets.

In future, TFTF can serve as a baseline model to initiate a multi-dimensional view on TFBS predictions. The model details are freely available to facilitate further advancements. We hope that our methods can lead to a better understanding of TFBS across cells and species, and can be further expanded to multiple epigenetic scenarios.

## Supplementary Material

btae013_Supplementary_Data

## Data Availability

The GRCh37 and mm10 reference genome was obtained from the NCBI database (https://www.ncbi.nlm.nih.gov). All the multiomic data can be found in the ENCODE Project website (https://www.encodeproject.org). We provided the ID of ENCODE experiments used in this study in Supplementary Table S1.

## Code availability

We provide a web server (https://tftf.ibreed.cn/) for users to utilize TFTF model. Users can train TFTF model and discover TFBS with their own data.
