# Supplementary material for: Multiomics-integrated deep language model enables in silico genome-wide detection of transcription factor binding site in unexplored biosamples
Source: Bioinformatics. 2024 Jan 12;40(1):btae013. doi: 10.1093/bioinformatics/btae013 (PMC10812877; doi:10.1093/bioinformatics/btae013)
Supplement: btae013_Supplementary_Data [file btae013_supplementary_data.zip › Extended_Data_Fig.1-4.pdf]

Extended Data Figures

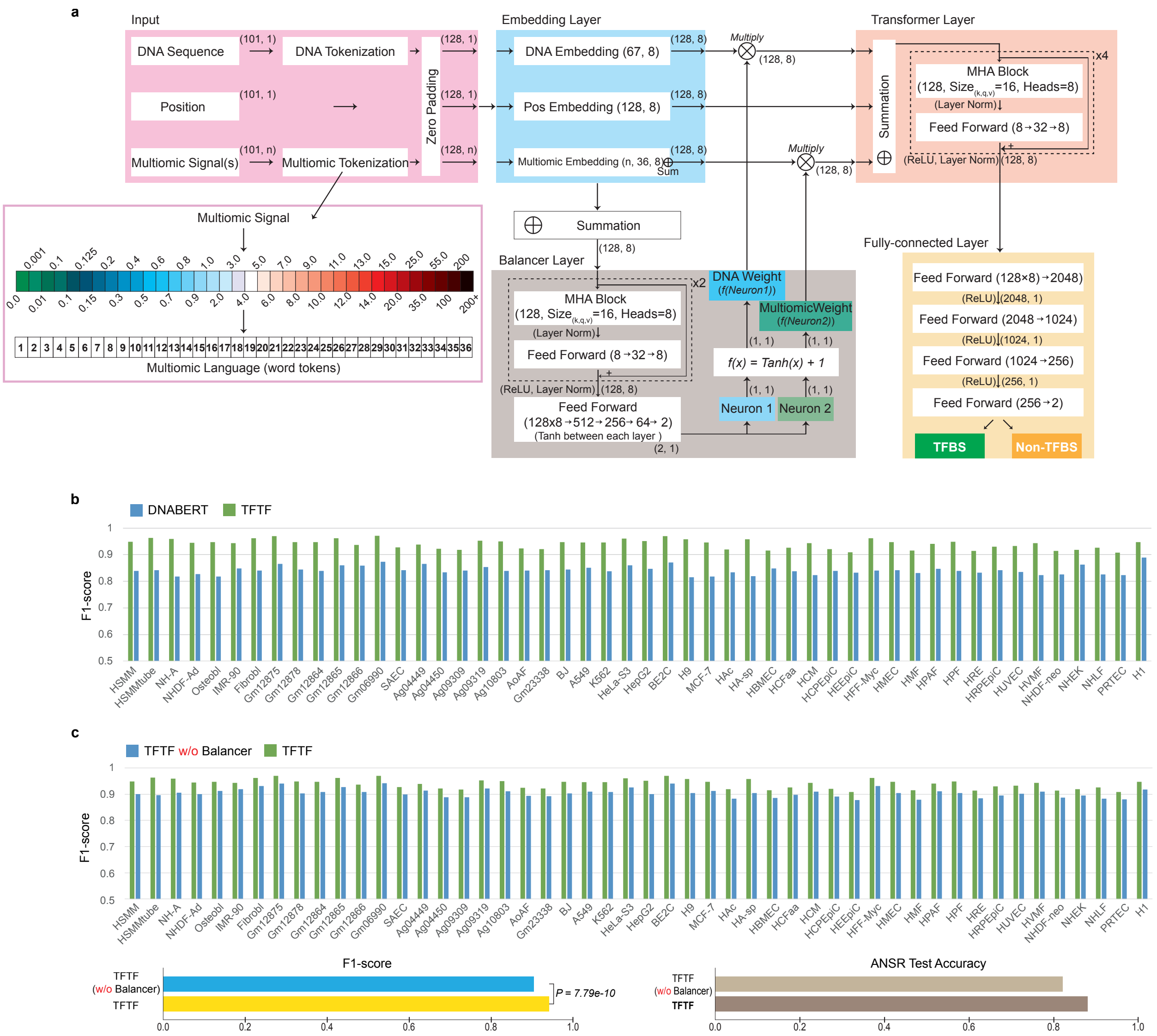

Extended Data Fig. 1. a. Detailed description of the architecture and parameters of the TFTF model as well as the output data shapes (represented as tuples on the right side of each block). The main trainable parameters are marked inside of white blocks. **b.** F1-score of 50 cell lines, predicted by TFTF (green bars) and DNABERT (blue bars). **c.** Ablation study of the TFTF model about the balancer layer. F1-scores of each of the 50 cell lines were calculated by TFTF with and without the balancer layer (green and blue bars, respectively). The mean F1-score and ANSR accuracy score of 50 cell lines were calculated accordingly (bottom horizontal bars), and *P* value was calculated by Wilcoxon Signed-Rank Test.

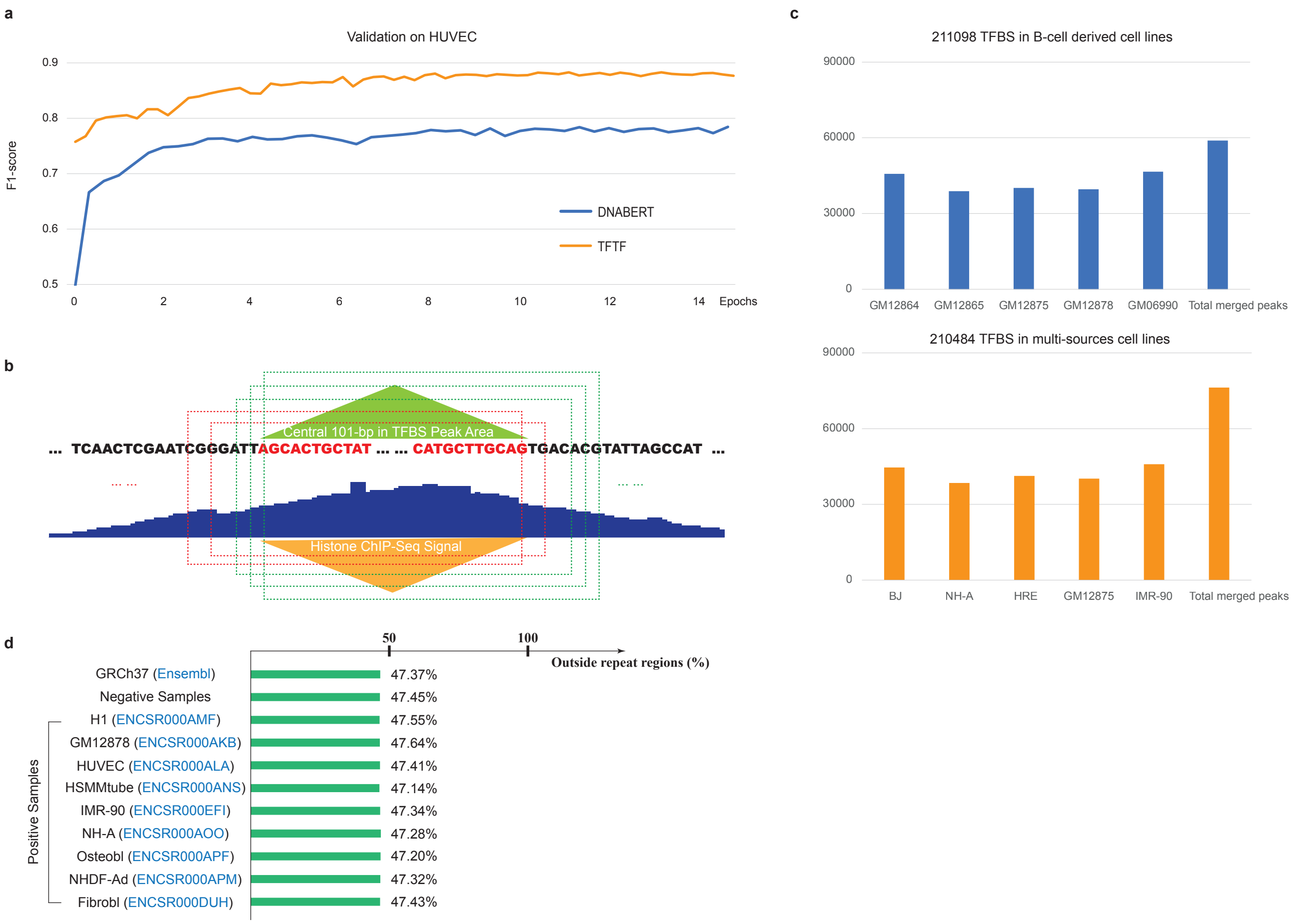

Extended Data Fig. 2. **a.** We plotted the F1-score of the validation dataset (HUVEC) at each epoch. The training process was early stopped when the F1-score converged (The F1-score of the next 4 consecutive epochs increased by less than 0.2% compared with the current epoch). **b.** Illustration of data augmentation. The TFBS peak region was shifted within $\pm$7-bp to both sides of the peak, resulting in a 14$\times$ larger dataset. **c.** The number of peaks in each B-cell derived cells, as well as the multiple cell types derived cells. The total number peaks between these two groups are comparable.

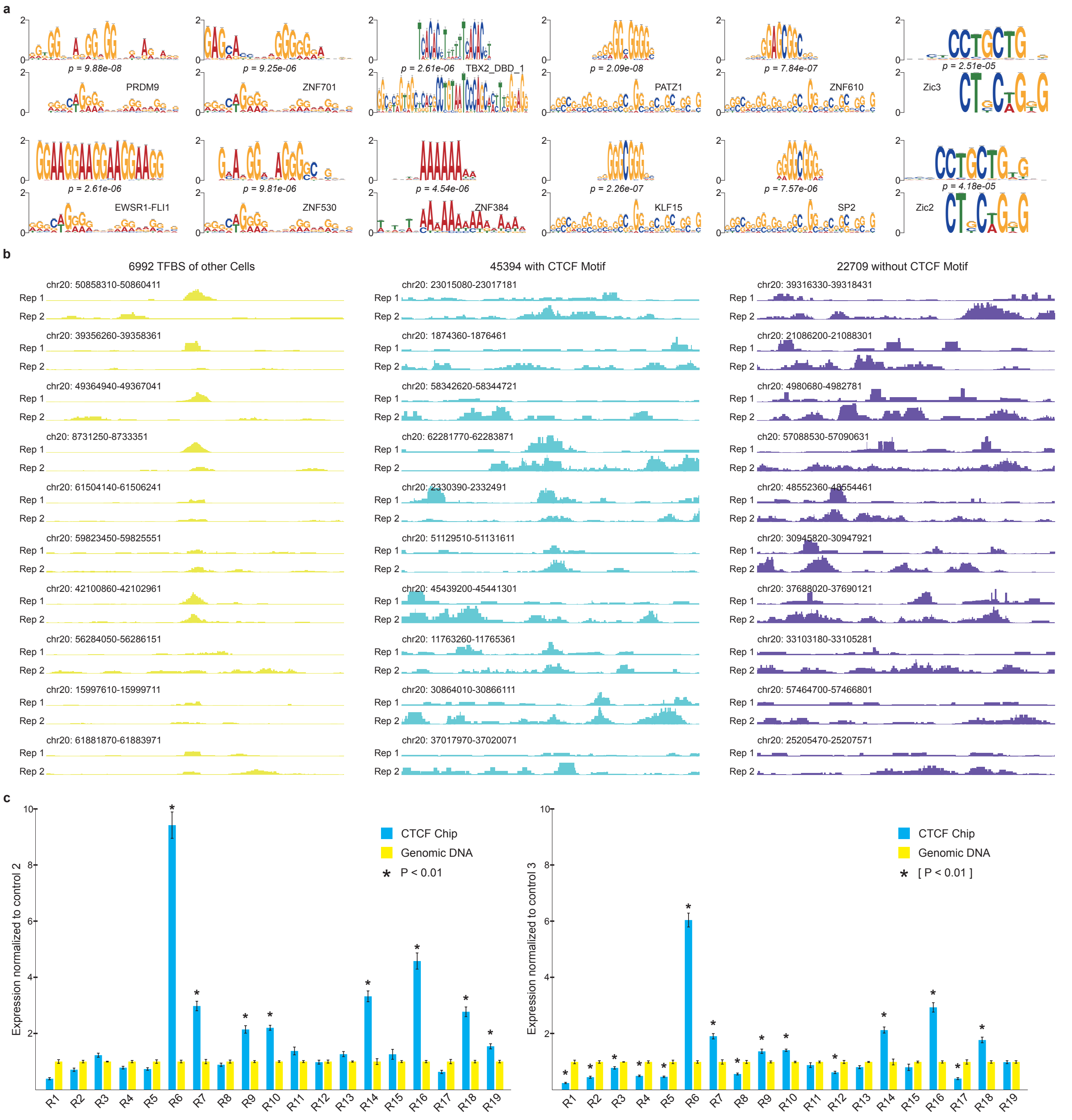

Extended Data Fig. 3. **a.** Top 5 de-novo motifs identified from FP predicted peaks were mapped to known human motif database. For the first 4 de-novo motifs, p-value $< 10^{-5}$ was used. For the last de-novo motif, top 2 mapped motifs of known TFs with the smallest p-values were shown. All the TFs names were marked accordingly. **b.** Top 10 signals of ChIP-seq peaks (in bigwig format) with highest Confidence Score were plotted. These included three types of FP samples: TFBS of non-H1 cells (yellow), TFBS with CTCF motif (cyan), and TFBS without CTCF motif (purple). Each region had two replicates. **c.** CTCF ChIP-qPCR experiments (**Methods**) were conducted in 19 regions (predicted as negative by IDR thresholded method, but positive by TFTF) and the expression level normalized to 2nd and 3rd control regions (genomic background with no CTCF ChIP signal) were plotted.

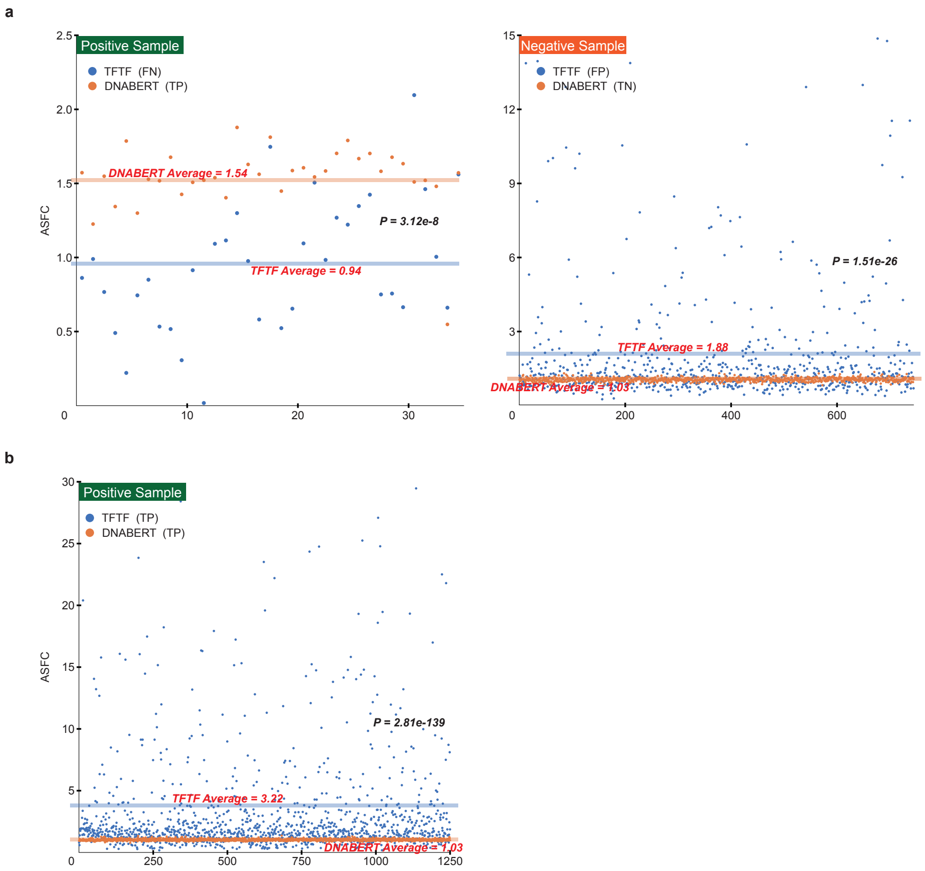

Extended Data Fig. 4. Attention score details in additional scenarios. Scattered dots illustrate the interplay between inside and outside CTCF motif regions as measured by ASFC. We utilized Wilcoxon Signed-Rank Test to calculate *P* values. **a.** ASFC of positive and negative samples where TFTF made incorrect predictions and DNABERT made correct predictions; and **b.** ASFC of positive samples which were recalled by both TFTF and DNABERT models.
